# Increased myocardial extracellular volume assessed by cardiovascular magnetic resonance T1 mapping and its determinants in type 2 diabetes mellitus patients with normal myocardial systolic strain

**DOI:** 10.1186/s12933-017-0651-2

**Published:** 2018-01-04

**Authors:** Yukun Cao, Wenjuan Zeng, Yue Cui, Xiangchuang Kong, Miao Wang, Jie Yu, Shan Zhang, Jing Song, Xu Yan, Andreas Greiser, Heshui Shi

**Affiliations:** 1 Department of Radiology, Union Hospital, Tongji Medical College, Huazhong University of Science and Technology, Wuhan, 430022 China; 2 Department of Clinical Laboratory, Union Hospital, Tongji Medical College, Huazhong University of Science and Technology, Wuhan, 430022 China; 3 MR Collaboration NE Asia, Siemens Healthcare, Shanghai, 201318 China; 4 Siemens Healthcare, Erlangen, Germany

**Keywords:** Diabetic cardiomyopathy, T1 mapping, Extracellular volume, Myocardial strain, Tissue tracking, Cardiac magnetic resonance

## Abstract

**Background:**

Cardiac magnetic resonance (CMR) T1 mapping and tissue-tracking strain analysis are useful quantitative techniques that can characterize myocardial tissue and mechanical alterations, respectively, in patients with early diabetic cardiomyopathy. The purpose of this study was to assess the left ventricular myocardial T1 value, extracellular volume fraction (ECV), and systolic strain in asymptomatic patients with type 2 diabetes mellitus (T2DM) and their underlying relationships with clinical parameters.

**Methods:**

We recruited 50 T2DM patients (mean age: 55 ± 7 years; 28 males) and 32 sex-, age-and BMI-matched healthy volunteers to undergo contrast-enhanced CMR examinations. The myocardial native T1, post-contrast T1 and ECV values of the left ventricle were measured from T1 and ECV maps acquired using the modified Look-Locker inversion recovery technique. The left ventricular global systolic strain and the strain rates were evaluated using routine cine images and tissue-tracking analysis software. The baseline clinical and biochemical indices were collected before the CMR examination.

**Results:**

The myocardial ECV and native T1 values were significantly higher in the diabetic patients than in the controls. (ECV: 27.4 ± 2.5% vs. 24.6 ± 2.2%, *p* < 0.001; native T1: 1026.9 ± 30.0 ms vs. 1011.8 ± 26.0 ms, *p* = 0.022). However, the left ventricular global systolic strain, strain rate, volume, myocardial mass, ejection fraction, and left atrial volume were similar between the diabetic patients and the healthy controls. In the diabetic patients, the native T1 values were independently correlated with the hemoglobin A1c levels (standardized *β* = 0.368, *p* = 0.008). The ECVs were independently associated with the hemoglobin A1c levels (standardized *β* = 0.389, *p* = 0.002), angiotensin-converting enzyme inhibitor (ACEI) treatment (standardized *β* = − 0.271, *p* = 0.025) and HCT values (standardized *β* = − 0.397, *p* = 0.001).

**Conclusions:**

Type 2 diabetes mellitus patients with normal myocardial systolic strain exhibit increased native T1 values and ECVs indicative of myocardial extracellular interstitial expansion, which might be related to poor glycemic control. The amelioration of myocardial interstitial matrix expansion might be associated with ACEI treatment. A valid assessment of the association of glucose control and ACEI treatment with myocardial fibrosis requires notably larger trials.

## Background

Over the last decade, cardiovascular disease has been the leading cause of morbidity and mortality for diabetic patients [1, 2]. Diabetic cardiomyopathy presenting with abnormal cardiac structure and function is associated with an increased risk of heart failure independent of hypertension or coronary artery disease [3, 4]. Multiple molecular mechanisms promote cardiomyocyte apoptosis and necrosis and diffuse myocardial fibrosis, ultimately leading to cardiac dysfunction and heart failure [5, 6]. Therefore, in myocardial fibrosis and dysfunction, early detection and intervention are essential for the prevention and management of diabetic cardiomyopathy.

Late gadolinium enhancement imaging with cardiovascular magnetic resonance (CMR) can accurately detect regional myocardial infarction or fibrosis [7]. However, this technology cannot identify diffuse myocardial fibrosis due to the absence of signal intensity differences between fibrosis and the normal myocardium [8]. CMR T1 mapping was recently shown to possibly be capable of overcoming this weakness through quantification of the T1 value and extracellular volume (ECV) within the myocardium as an imaging marker to assess the myocardial interstitial matrix [9]. Previous studies in diabetic patients and animals have indicated the presence of diffuse myocardial fibrosis based on histological evidence [10, 11]. Some studies also revealed that diabetic patients had lower post-contrast myocardial T1 values and greater ECVs, suggestive of extracellular interstitial expansion compared with healthy subjects, although the left ventricular volumes and ejection fractions were normal [12, 13]. However, another study observed no difference in the myocardial ECVs between diabetic patients and healthy controls [14]. Additionally, previous publications reported that abnormal myocardial T1 or ECV values were associated with diastolic dysfunction, systolic strain impairment, and metabolic disturbances [15, 16]. However, whether abnormal T1 and ECV values exist in asymptomatic preclinical diabetic cardiomyopathy patients and the associations of these values with cardiac functions and the involved clinical indices remain unclear.

Previous research using speckle tracking echocardiography (STE) has revealed myocardial systolic strain dysfunction in diabetic patients [17–21]. However, whether global longitudinal, radial or circumferential strain is reduced in patients compared with healthy controls is controversial. For example, some studies have demonstrated that patients exhibit a lower global longitudinal strain than healthy subjects [18, 20]. Nevertheless, a large study reported that there was no difference in the longitudinal strain between patients without albuminuria and controls [21]. Additionally, the use of the STE method to assess myocardial strain has several limitations, including a lower signal-to-noise ratio, weak acoustic windows, and through-plane motion artifacts [22, 23]. Furthermore, CMR tissue-tracking technology can assess cardiac deformation parameters with post-processing for cine images, which have a higher signal-to-noise ratio, and utilizes routine cine images without requiring any additional scan time [22, 24]. Currently, the CMR tissue-tracking technique has been increasingly used for myocardial strain evaluation in different types of cardiomyopathy due to the simple application and rapid post-processing advantages of this method [23, 25–27].

Consequently, we used multiple advanced CMR technologies, including T1 mapping and tissue-tracking analysis, to compare the myocardial tissue characterization and strain alterations between patients with type 2 diabetes mellitus (T2DM) and healthy volunteers and to assess the contribution of related factors to early diabetic cardiomyopathy.

## Methods

### Study population

We prospectively recruited 50 patients with T2DM from the endocrine department of Wuhan Union Hospital and 32 healthy volunteers matched by age, sex and BMI from the community. The inclusion criteria for the T2DM patients were clinically confirmed subjects in accordance with the World Health Organization criteria [28] aged between 30 and 70 years with no history of heart disease (congenital heart disease, coronary artery disease, cardiomyopathy or valvular heart disease), no clinical manifestations presenting with chest pain, palpitation, or dyspnea and normal electrocardiogram findings. The inclusion criteria for the controls were an age > 30 years, no history of hypertension, hyperlipidemia, diabetes mellitus, or heart disease, a normal physical examination and normal electrocardiogram findings. The exclusion criteria included renal dysfunction (glomerular filtration rate < 30 mL/min), active pregnancy, contraindications for MRI (implanted metallic objects, such as a cardiac pacemaker, metal valve and insulin pump, or an allergy to contrast media), diabetes with poorly controlled hypertension and left ventricular hypertrophy (a left ventricular myocardial mass from CMR imaging indexed by a body surface area greater than 61 g/m^2^ for women or 81 g/m^2^ for men according to the definition proposed by Olivotto et al. [29]), and the presence of abnormal cardiac dimensions, global or regional left ventricular wall motion abnormalities, valvular stenosis or regurgitation or myocardial late gadolinium enhancement (LGE) during the MRI examination. This study was approved by the ethics committee of Tongji Medical College of Huazhong University of Science and Technology. All subjects who participated in this study signed written informed consent forms after receiving detailed information about the research.

### Anthropometric and biochemistry

The sex, age, height, body weight, and blood pressure of all subjects were recorded. Fasting blood samples were obtained at the time of the MRI examination. The hematocrit (HCT) was measured to calculate the ECV of the myocardium. Other biochemical indices, including serum glucose, hemoglobin A1c (HbA1C), creatinine, blood urea nitrogen (BUN), total cholesterol, triglycerides, high-density lipoprotein cholesterol (HDL-C) and low-density lipoprotein cholesterol (LDL-C), were obtained from the patients with T2DM.

### CMR scanning protocol

Cardiac magnetic resonance imaging was performed using a 1.5T MR scanner (MAGNETOM Area, Siemens Healthcare, Erlangen, Germany) with vector-electrocardiographic gating and an 18-channel phased array surface coil in combination with a 32-element spine array coil. A balanced steady-state free procession (b-SSFP) sequence was performed to acquire left ventricular long-axis and short-axis (coverage from the base to the apex segment) cines. The cine acquisition parameters were as follows: repetition time, 2.93 ms; echo time, 1.16 ms; flip angle, 80°; slice thickness, 6 mm; field of view, 340 × 255 mm^2^; and matrix, 256 × 205.

Native T1 maps were performed at the basal, mid and apical slices of the left ventricular short axis using a prototype-modified Look-Locker inversion recovery (MOLLI) sequence. The T1 mapping acquisition parameters were as follows: repetition time, 3.89 ms; echo time, 1.12 ms; flip angle, 35°; slice thickness, 8 mm; field of view, 360 × 270 mm^2^; matrix, 256 × 192; PAT factor, 2; and acquisition scheme, 5b(3b)3b.

Delayed-enhancement imaging of the long- and short-axes for the left ventricle was performed 10 min after a bolus injection of intravenous gadolinium-diethylenetriamine pentaacetic acid (DTPA) (0.2 mmol/kg, Magnevist; Bayer Healthcare; Germany) with a phase-sensitive inversion recovery (PSIR) sequence. The LGE imaging parameters were as follows: repetition time, 12.44 ms; echo time, 1.19 ms; inversion recovery time, 300 ms; flip angle, 40°; slice thickness, 8 mm; field of view, 360 × 270 mm^2^; and matrix, 256 × 192.

Fifteen minutes after contrast administration, post-contrast T1 maps were acquired at the same level used for the native T1 mapping. The mapping acquisition parameters were as follows: repetition time, 3.89 ms; echo time, 1.12 ms; flip angle, 35°; slice thickness, 8 mm; field of view, 360 × 270 mm^2^; matrix, 256 × 192; PAT factor, 2; and acquisition scheme, 4b(1b)3b(1b)2b.

### Assessment of cardiac volume and function

Commercial post-processing software (cvi^42^, Circle Cardiovascular imaging, Calgary, AB, Canada) was used to analyze the cardiac structure and function offline. After all short-axis cine images were imported into this software, we manually outlined the left ventricular endocardium and epicardium on all contiguous short-axis cine images. The trabeculae and papillary muscles were included in the left ventricular cavity. Next, the left ventricular end-diastolic volume (LVEDV), end-systolic volume (LVESV), stroke volume (LVSV), ejection fraction (LVEF) and mass (LVM) were measured. Additionally, the left atrial volume (LAV) was calculated according to the biplane area-length method (LAV = [0.85 × (2-chamber area) × (4-chamber area)]/L, where L was the shortest dimension from the back wall to the line across hinge points of the mitral valve between the above two chambers) [30]. All of the above measurements were indexed to the body surface area (BSA).

### Measurement of T1 mapping and ECV mapping

**Fig. 1:**
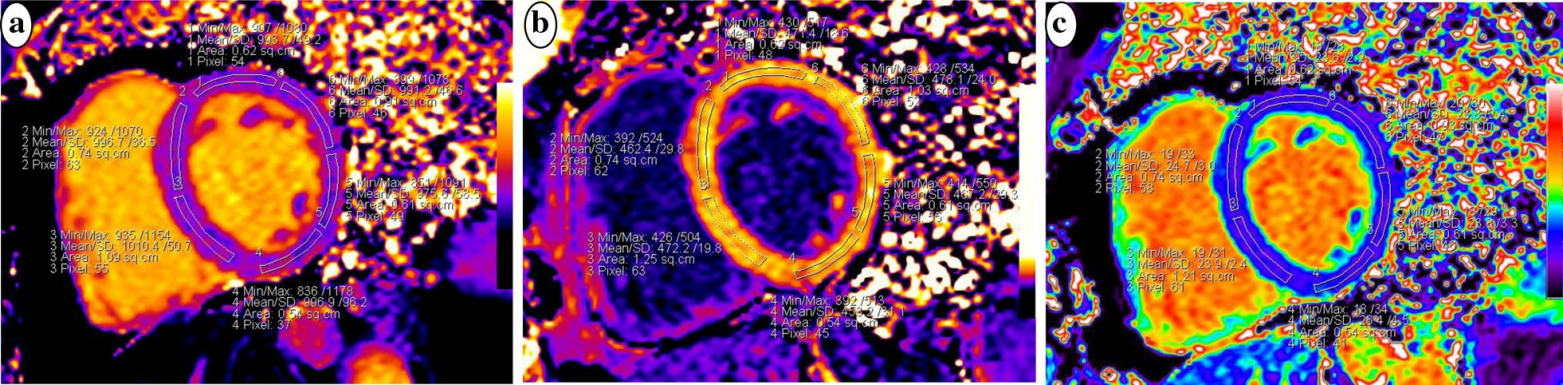
Representative maps of a healthy volunteer at the left ventricular middle short-axis segment with a modified Look-Locker inversion recovery (MOLLI) sequence showing native T1 mapping (**a**), post-contrast T1 mapping of the same slice (**b**), and calculated extracellular volume (ECV) mapping of the same segment (**c**)

Extracellular volume fraction mapping was automatically calculated using a prototype inline processing function from Siemens, based on native and post-contrast T1 mapping at the same slice and the subject-specific HCT value. The native and post-contrast T1 and ECV values were measured by manually delineating regions of interest in the mid-layer myocardium of the left ventricular basal, middle and apical segments. The 16 regions of interest for each volunteer were drawn based on the American Heart Association 16-segment model [31] (Fig. 1). Any segments with artifacts affecting the measurements were eliminated. Next, the global mean T1 and ECV values of each subject were calculated. To determine the reproducibility of myocardial T1 and ECV, the same images from 15 randomly selected individuals were repeatedly measured by the same observer and another blinded observer.

### Measurement of myocardial systolic strain

**Fig. 2:**
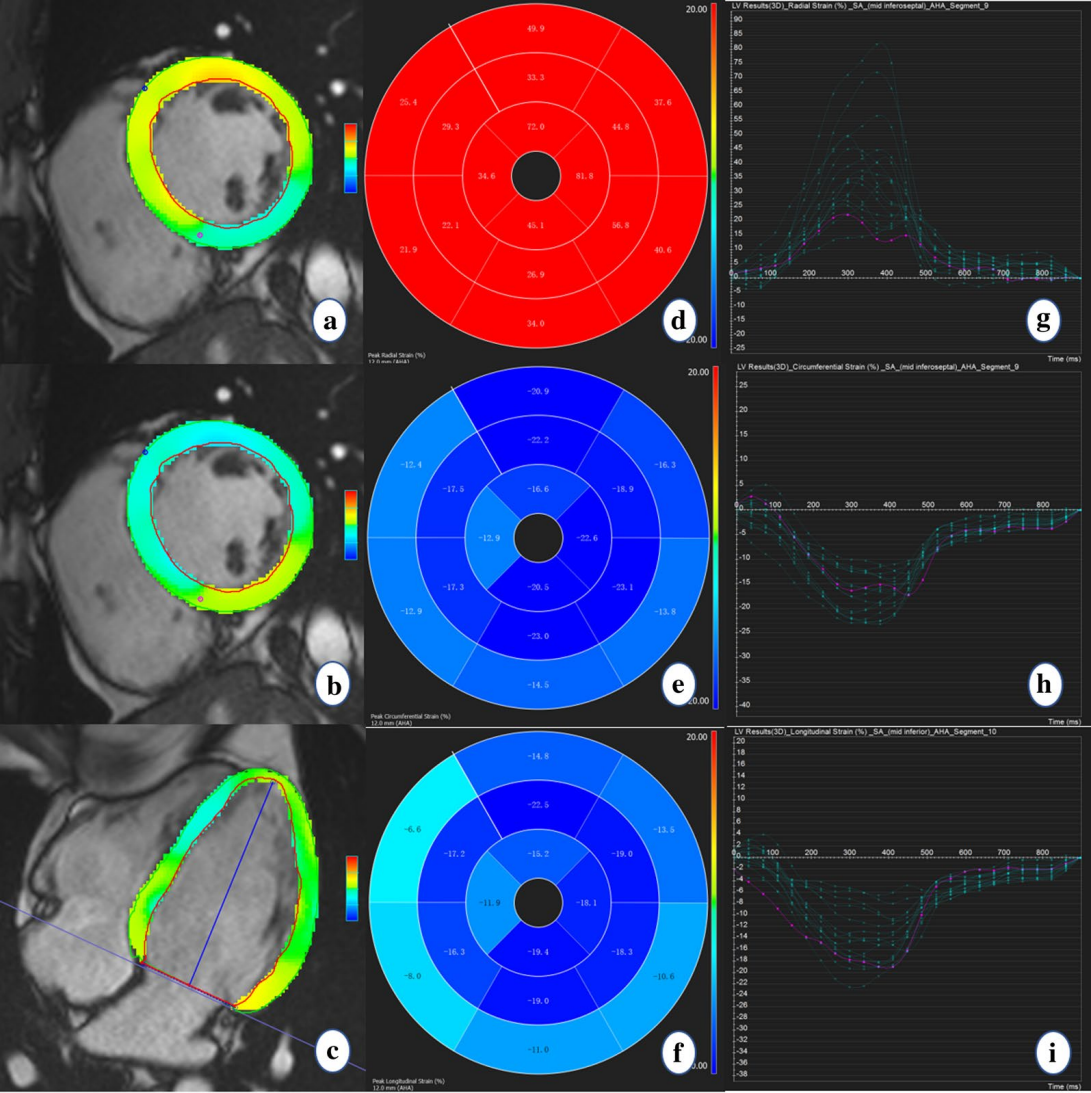
Diagram of the peak systolic strain analysis of the left ventricular myocardium in a healthy volunteer. The colored tissue-tracking maps of radial (**a**), circumferential (**b**), and longitudinal (**c**) strain analysis are presented on the left. The radial (**d**), circumferential (**e**), and longitudinal (**f**) strain values in a 16-segment model are displayed in the middle. The radial (**g**), circumferential (**h**), and longitudinal (**i**) strain–time curves in a cardiac cycle are presented on the right

Tissue tracking was performed off-line using dedicated commercial software (cvi^42^, Circle, Calgary, AB, Canada). A consecutive stack of short-axis cine images and 2- and 4-chamber long-axis images were imported into this software. The left ventricular endocardial and epicardial contours of the end diastole were manually delineated on the short-axis and two long-axis cine images. The trabeculae and papillary muscles were included in the left ventricular cavity. The left ventricular global longitudinal (GLS), circumferential (GCS) and radial strain (GRS) and the early systolic strain rate were calculated by automatically tracking the contours in each cardiac cycle (Fig. 2). To determine the reproducibility of the myocardial strain measurement, the same images from 15 randomly selected individuals were repeatedly measured by the same observer and another blinded observer.

### Statistical analysis

Statistical analyses of all data were performed using SPSS software (SPSS 21.0 for Windows, IBM, Chicago, IL, USA). The normality of all continuous data was checked using the Kolmogorov–Smirnov test. Normally and non-normally distributed data and categorical variables are expressed as the means ± standard deviations and the medians (interquartile range) and frequencies (percentages), respectively. The independent-sample Student’s t test was used to compare two groups of normally distributed variables, and the Chi square test was used to compare categorical variables. The analysis of covariance (ANCOVA) was used to compare HCT-adjusted ECV values between two groups. Correlational analyses were performed with Pearson’s correlation for normally distributed variables and Spearman’s correlation for non-normally distributed data. Multiple linear regression analyses were used to identify the determinants of the myocardial T1 values and ECVs in patients with T2DM. All candidate variables (*p* < 0.2 in the univariate linear regression analysis and without collinearity) were selected for entry into the multiple stepwise regression model. A *p* value < 0.05 (two-tailed) was considered significant.

## Results

### Clinical characteristics of the study population

Table 1 presents the baseline clinical data for the 50 T2DM patients (age: 55 ± 7 years, 28 males, BMI: 24.7 ± 3.7 kg/m^2^) and 32 controls (age: 54 ± 6 years, 17 males, BMI: 23.7 ± 2.3 kg/m^2^). The median diabetic duration and mean HbA1C in the patients were 10 years (IQR: 6–13) and 8.9 ± 2.3%, respectively. No differences were observed with respect to sex, age, blood pressure or BMI between the patients and the controls. Of the 50 diabetic patients, 19 had well-controlled hypertension, 14 had retinopathy, 10 had neuropathy and 6 had peripheral vascular disease.

**Table 1:** Clinical characteristics of the study population

	T2DM (n = 50)	Control (n = 32)	*p* value
Age (year)	55 ± 7	54 ± 6	0.483
Male (n, %)	28 (56)	17 (53.1)	0.799
BMI (kg/m^2^)	24.7 ± 3.7	23.7 ± 2.3	0.165
Diabetes duration (year)	10 (6–13)	–	–
HR (bpm)	69 ± 9	66 ± 9	0.128
SBP (mmHg)	128 ± 14	124 ± 10	0.215
DBP (mmHg)	78 ± 10	75 ± 8	0.159
Hypertension (n, %)	19 (38)	–	–
Diabetic complication (n, %)			
Retinopathy	14 (28)	–	–
Neuropathy	10 (20)	–	–
Peripheral vascular disease	6 (12)	–	–
Hematocrit (%)	39.3 ± 4.1	41.7 ± 3.7	0.009*
BUN (mmol/L)	5.3 ± 1.6	–	–
Creatinine (μmol/L)	68.9 ± 14.7	–	–
Total cholesterol (mmol/L)	4.4 ± 1.0	–	–
Triglycerides (mmol/L)	1.62 ± 1.2	–	–
HDL-C (mmol/L)	1.3 ± 0.3	–	–
LDL-C (mmol/L)	2.5 ± 0.7	–	–
FPG (mmol/L)	8.3 ± 3.3	–	–
Hemoglobin A1C (%)	8.9 ± 2.3	–	–
Hypoglycemic medication, n (%)			
Insulin	24 (48)	–	–
Metformin	22 (44)	–	–
Sulfonylurea	10 (20)	–	–
Other medication, n (%)			
Statin	21 (42)	–	–
Aspirin	16 (32)	–	–
ACEI	20 (40)	–	–
Diuretics	5 (10)	–	–
Calcium channel blockers	5 (10)	–	–
β-blockers	9 (18)	–	–

All data are expressed as the means ± SDs, percentages (numbers of participants), or medians (interquartile ranges) as appropriate

*DM* diabetes mellitus, *BMI* body mass index, *HR* heart rate, *SBP* systolic blood pressure, *DBP* diastolic blood pressure, *BUN* blood urea nitrogen, *HDL-C* high-density lipoprotein cholesterol, *LDL-C* low-density lipoprotein cholesterol, *FPG* fasting plasma glucose, *ACEI* angiotensin-converting enzyme inhibitor

* *p* < 0.05 between groups

### MRI characteristics of the study population

Table 2 presents the baseline MRI characteristics of the patients with T2DM and the controls. No significant differences were observed in the LVEDV index (61.0 ± 12.6 vs. 63.7 ± 11.2 mL/m^2^; *p* = 0.336), LVESV index (28.3 ± 6.7 vs. 27.5 ± 6.4 mL/m^2^; *p* = 0.611), LVEF (55.0 ± 6.2 vs. 57.2 ± 4.6%; *p* = 0.077), LVM index (53.9 ± 8.7 vs. 55.9 ± 6.6 g/m^2^; *p* = 0.269) and LAV index (38.1 ± 10.0 vs. 36.9 ± 11.0 mL/m^2^; *p* = 0.585) between the patients and the controls.

**Table 2:** MRI characteristics of the study population

	T2DM (n = 50)	Control (n = 32)	*p* value
LVEDV index (mL/m^2^)	61.0 ± 12.6	63.7 ± 11.2	0.336
LVESV index (mL/m^2^)	28.3 ± 6.7	27.5 ± 6.4	0.611
LVEF (%)	55.0 ± 6.2	57.2 ± 4.6	0.077
LVM index (g/m^2^)	53.9 ± 8.7	55.9 ± 6.6	0.269
LAV index (mL/m^2^)	38.1 ± 10.0	36.9 ± 11.0	0.585
Native T1 (ms)	1026.9 ± 30.0	1011.8 ± 26.0	0.022*
Post-contrast T1 (ms)	460.2 ± 24.7	459.9 ± 26.1	0.967
ECV (%)	27.4 ± 2.5	24.6 ± 2.2	< 0.001*
HCT-adjusted ECV (%)	27.1 ± 0.30^a^	25.0 ± 0.38^a^	< 0.001*
LVGRS (%)	44.8 ± 10.5	43.7 ± 11.4	0.650
LVGRSR-S (1/s)	2.6 ± 0.9	2.6 ± 1.0	0.868
LVGCS (%)	− 19.3 ± 2.4	− 19.7 ± 2.7	0.540
LVGCSR-S (1/s)	− 0.9 ± 0.2	− 1.0 ± 0.2	0.696
LVGLS (%)	− 17.3 ± 2.2	− 17.4 ± 2.2	0.861
LVGLSR-S (1/s)	− 0.8 ± 0.2	− 0.9 ± 0.2	0.764

*DM* diabetes mellitus, *LVEDV* left ventricular end-diastolic volume, *LVESV* left ventricular end-systolic volume, *LVEF* left ventricular ejection fraction, *LVM* left ventricular mass, *LAV* left atrium volume, *ECV* extracellular volume, *HCT* hematocrit, *GRS* global radial strain, *GRSR-S* peak systolic global radial strain rate, *GCS* global circumferential strain, *GCSR-S* peak systolic global circumferential strain rate, *GLS* global longitudinal strain, *GLSR-S* peak systolic global longitudinal strain rate

* *p* < 0.05 between groups

^a^These data are expressed as the means ± SEs, whereas all other data are expressed as the means ± SDs

**Fig. 3:**
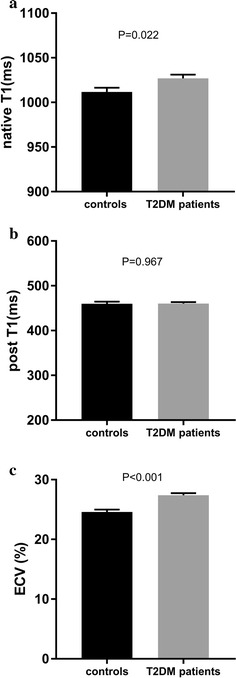
Comparison of the mean native myocardial T1 (**a**), post-contrast T1 (**b**) and ECV (**c**) values between the healthy controls and T2DM patients. *ECV* extracellular volume fraction, *T2DM* type 2 diabetes mellitus

Measurement of the T1 and ECV mapping demonstrated that the mean native T1 value (1026.9 ± 30.0 ms vs. 1011.8 ± 26.0 ms; *p* = 0.022) and ECV (27.4 ± 2.5% vs. 24.6 ± 2.2%; *p*<0.001) were significantly higher in the diabetic patients than in the healthy controls. The ANCOVA indicated that the HCT-adjusted ECV value was also significantly higher in the diabetic patients (27.1 ± 0.30%) than in the healthy controls (25.0 ± 0.38%) (*F* = 17.525, *p* < 0.001). However, no difference in the post-contrast T1 values (460.2 ± 24.7 ms vs. 459.9 ± 26.1 ms; *p* = 0.967) was observed between the patients and the controls (Fig. 3).

The myocardial systolic strain assessment revealed no significant differences in the left ventricular global radial (44.8 ± 10.5 vs. 43.7 ± 11.4%; *p* = 0.650), circumferential (− 19.3 ± 2.4 vs. − 19.7 ± 2.7%; *p* = 0.540) and longitudinal strain (− 17.3 ± 2.2 vs. − 17.4 ± 2.2%; *p* = 0.861) between the diabetic patients and the controls. Similarly, there were no significant differences in the left ventricular global systolic radial (2.6 ± 0.9 vs. 2.6 ± 1.0 ^1^/s; *p* = 0.868), circumferential (− 0.9 ± 0.2 vs. − 1.0 ± 0.2 ^1^/s; *p* = 0.696) and longitudinal strain rate (− 0.8 ± 0.2 vs. − 0.9 ± 0.2 ^1^/s; *p* = 0.764) between the two groups (Table 2).

### Factors associated with native T1 values and ECV in diabetic patients

Table 3 summarizes the univariate correlation coefficients between the mean myocardial native T1 values, ECV, and baseline clinical characteristics and the MRI characteristics in the diabetic patients.

**Table 3:** Univariate correlation coefficients for native T1 and ECV in diabetic patients

Variable	Native T1 (ms)		ECV (%)	
	r value	*p* value	r value	*p* value
Age (year)	− 0.135	0.351	− 0.129	0.37
HR (bpm)	0.058	0.689	0.149	0.301
BMI (kg/m^2^)	0.002	0.989	− 0.26	0.068
Diabetes duration (year)	0.006^a^	0.969^a^	− 0.076^a^	0.599^a^
SBP (mmHg)	− 0.09	0.533	− 0.241	0.091
DBP (mmHg)	− 0.221	0.124	− 0.252	0.078
Retinopathy	0.217	0.129	− 0.085	0.558
Neuropathy	0.131	0.364	0.134	0.355
Peripheral vascular disease	0.014	0.924	0.015	0.918
LVEDV index (mL/m^2^)	− 0.167	0.248	0.174	0.227
LVESV index (mL/m^2^)	− 0.207	0.149	0.154	0.286
LVEF (%)	0.16	0.267	− 0.124	0.391
LVM index (g/m^2^)	0.088	0.544	− 0.197	0.171
LAV index (mL/m^2^)	− 0.082	0.571	0.112	0.438
LVGRS (%)	− 0.075	0.607	− 0.159	0.269
LVGRSR-S (1/s)	− 0.038	0.795	− 0.086	0.554
LVGCS (%)	− 0.112	0.439	− 0.041	0.779
LVGCSR-S (1/s)	− 0.141	0.33	− 0.102	0.482
LVGLS (%)	0.049	0.736	0.114	0.431
LVGLSR-S (1/s)	− 0.086	0.55	− 0.108	0.455
BUN (mmol/L)	− 0.122	0.4	0.085	0.559
Creatinine (μmol/L)	− 0.191	0.185	− 0.244	0.088
Total cholesterol (mmol/L)	0.025	0.866	0.059	0.682
Triglycerides (mmol/L)	0.14	0.332	− 0.112	0.437
Hematocrit (%)	− 0.055	0.704	− 0.370	0.008*
HDL-C (mmol/L)	− 0.1	0.49	0.164	0.256
LDL-C (mmol/L)	− 0.076	0.599	0.022	0.879
FPG (mmol/L)	0.172	0.232	0.107	0.458
Hemoglobin A1C (%)	0.368	0.008*	0.430	0.002*
Insulin	− 0.018	0.902	− 0.051	0.726
Metformin	0.217	0.13	− 0.07	0.627
Sulfonylurea	− 0.019	0.894	0.121	0.401
Statin	− 0.085	0.557	− 0.082	0.572
Aspirin	− 0.128	0.374	− 0.078	0.592
ACEI	− 0.05	0.728	− 0.359	0.011*
Diuretics	− 0.129	0.372	− 0.125	0.386
Calcium channel blockers	0.195	0.176	− 0.063	0.664
β-Blockers	− 0.037	0.797	− 0.045	0.754

*ECV* extracellular volume, *HR* heart rate, *BMI* body mass index, *SBP* systolic blood pressure, *DBP* diastolic blood pressure, *LVEDV* left ventricular end-diastolic volume, *LVESV* left ventricular end-systolic volume, *LVEF* left ventricular ejection fraction, *LVM* left ventricular mass, *LAV* left atrial volume, *GRS* global radial strain, *GRSR-S* peak systolic global radial strain rate, *GCS* global circumferential strain, *GCSR-S* peak systolic global circumferential strain rate, *GLS* global longitudinal strain, *GLSR-S* peak systolic global longitudinal strain rate, *BUN* blood urea nitrogen, *HDL-C* high-density lipoprotein cholesterol, *LDL-C* low-density lipoprotein cholesterol, *FPG* fasting plasma glucose, *ACEI* angiotensin-converting enzyme inhibitor

* *p* < 0.05 between groups

^a^These data were analyzed using Spearman’s correlation, all other data were analyzed using Pearson’s correlation

**Fig. 4:**
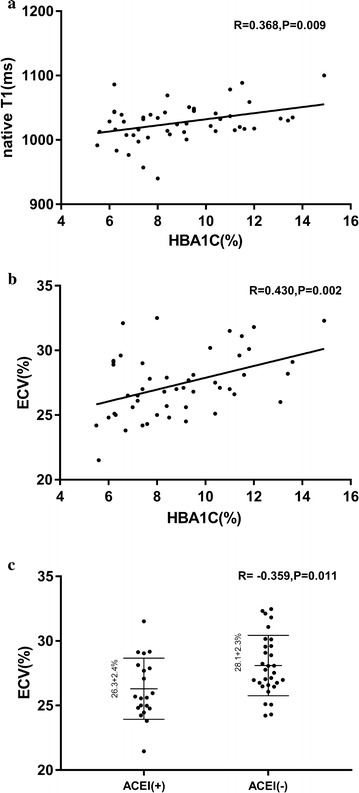
**a** Relationship between the native myocardial T1 values and the HbA1C levels in the diabetic patients; **b** relationship between the myocardial ECV values and the HbA1C levels in the diabetic patients; **c** relationship between the myocardial ECV values and ACEI treatment in the diabetic patients. *ECV* extracellular volume fraction, *HbA1C* hemoglobin A1c, *ACEI* angiotensin-converting enzyme inhibitor

In the patient group, the mean native T1 values were significantly associated with the HbA1C levels (r = 0.368, *p* = 0.008) (Fig. 4). However, no significant associations were observed between the native T1 values and the age, heart rate, BMI, DM duration, heart volume and function parameters, other biochemical indices, diabetic complications, or medications. In the multivariable stepwise analysis, the independent determinant of the native T1 value was the HbA1C value (*β* = 0.368, *p* = 0.008, model R^2^ = 0.136) (Table 4).

**Table 4:** Independent determinants of native T1 and ECV in diabetic patients

Variable	Unstandardized *β*	Standardized *β*	*p* value
Native T1			
Hemoglobin A1C	4.735	0.368	0.008
ECV			
Hemoglobin A1C	0.413	0.389	0.002
Hematocrit	− 0.239	− 0.397	0.001
ACEI	− 1.358	− 0.271	0.025

*ECV* extracellular volume, *ACEI* angiotensin-converting enzyme inhibitor

Additionally, the ECV values for the diabetic patients were significantly correlated with the HbA1C level (r = 0.430, *p* = 0.002), angiotensin-converting enzyme inhibitor (ACEI) treatment (r = − 0.359, *p* = 0.011) and HCT values (r = − 0.370, *p* = 0.008) (Fig. 4). However, no significant associations were detected between the ECV values and the age, heart rate, BMI, DM duration, heart volume and function parameters, other biochemical indices, diabetic complications or medications. In the multivariable analysis, the independent determinants of the ECV value were HbA1C (*β* = 0.389, *p* = 0.002), ACEI treatment (*β* = − 0.271, *p* = 0.025) and HCT values (*β* = − 0.397, *p* = 0.001) (model R^2^ = 0.412) (Table 4).

### Intra-observer and inter-observer reproducibility

The intra-class correlation coefficient (ICC) values in the intra-observer analysis were 0.967, 0.980, 0.984, 0.965, 0.974, and 0.899 for native T1, post-contrast T1, ECV, GRS, GCS, and GLS, respectively. The ICC values in the interobserver analysis were 0.972, 0.971, 0.975, 0.973, 0.963, and 0.893 for native T1, post-contrast T1, ECV, GRS, GCS, and GLS, respectively.

## Discussion

The present study demonstrated that patients with T2DM exhibited significantly increased mean native T1 values and ECVs of the LV myocardium compared with the normal control group, although no significant differences in the LV structural and functional parameters were observed between the two groups. Moreover, the increased myocardial native T1 values and ECVs were significantly associated with poor glycemic control (HbA1c), and ACEI treatment was related to a lower myocardial ECV.

Cardiac magnetic resonance T1 mapping to quantify diffuse myocardial fibrosis has been validated in many previous histological studies [32–36]. These studies demonstrated that the native myocardial T1 values [35, 36] and ECVs [32, 35, 36] were significantly positively associated with the histological collagen volume fraction. Therefore, our findings suggest that diabetic patients with higher native T1 and ECV values relative to healthy controls might develop cardiac interstitial fibrosis. Previous studies have indicated that T2DM patients [37, 38] exhibit increased myocardial ECVs compared with healthy subjects, which is consistent with our present study findings. However, Levelt et al. [14] announced that the difference in the myocardial ECVs between middle-aged T2DM patients and healthy subjects was not significant. This discrepancy may be due to the stricter inclusion criteria applied in Levelt’s study. Specifically, diabetic patients with insulin administration, an HbA1c level > 9%, or accompanying diabetic complications were excluded, whereas other studies, including ours, did not exclude these patients [37, 38]. Additionally, the native T1 value, which is an integrated signal of cardiomyocytes and the interstitial matrix, was increased in the present of edema or expansion of the interstitial space [39]. The current study demonstrated that the native T1 value was higher in diabetic patients with an increased myocardial ECV than in healthy volunteers, which was consistent with Vasanji’s study [13]. Thus, in future research, native T1 mapping might be performed to preliminarily detect myocardial extracellular interstitial expansion in diabetic patients with end-stage renal disease, although contrast-enhanced MRI is contraindicated. Furthermore, previous studies have indicated that the post-contrast T1 values are significantly lower in diabetic patients than in healthy controls and inversely correlated with myocardial diastolic function [12, 15]. However, our study did not observe decreased post-contrast T1 values in diabetic patients. One possible explanation for this difference is that multiple factors affect the post-contrast T1 values, including the contrast media dosing, acquisition time after contrast agent administration, renal clearance rate, and contrast agent transfer rate between the extracellular matrix and microvasculature [40, 41]. Accordingly, native myocardial T1 and ECV might be relatively more sensitive parameters for detecting expansion of the myocardial extracellular matrix in diabetic patients compared with the post-contrast T1 value.

In the present study, the MR tissue-tracking-derived strain analysis demonstrated that the LV global systolic longitudinal, circumferential and radial strain and the strain rates were similar between diabetic patients and healthy individuals. Significant alterations in the LV and LA volumes and the LV mass were not observed. Therefore, native T1 and ECV, as early parameters, may be more sensitive than the myocardial systolic strain for the detection of early diabetic cardiomyopathy given the increased native T1 values and ECVs of the diabetic patients in our study. However, we did not observe decreased myocardial systolic strain as reported in some previous studies [18, 19, 21, 42]. Moreover, whether myocardial systolic strain decreases in patients with early diabetic cardiomyopathy is controversial. Studies using ultrasound speckle tracking [18, 19] or MR displacement encoding with stimulated echoes (DENSE) [42] have suggested that diabetic patients exhibit decreases in the longitudinal, circumferential or radial strain compared with control groups. However, Jensen et al. [21] indicated that the myocardial systolic strain was normal in diabetic patients without albuminuria in a large sample study. The above differences in the myocardial strain measurements may be due to differences in the study populations and different strain acquisition methods. Specifically, the mean diabetic duration was more than 20 years in Jensen’s study [21], whereas the duration in other studies [18, 19, 42], including ours, was only approximately one decade. Furthermore, Jensen’s study [21] focused primarily on T1DM patients, whereas other studies [19, 42], including ours, focused on T2DM patients. There may be some differences between T1DM and T2DM, including age of disease onset, cardiovascular risk factors, complications and clinical medications [1], which may lead to different levels of cardiac abnormalities. Third, our strain acquisition method was MR-derived tissue tracking technology, whereas other studies employed MR-DENSE [42] and ultrasound speckle tracking [18, 19, 21]. The present study indicated that the increased ECV indicative of myocardial interstitial fibrosis might not lead to myocardial strain abnormalities in early diabetic cardiomyopathy. Therefore, the severity of myocardial fibrosis in this study may not be sufficient to cause early myocardial systolic dysfunction. Additionally, the increased ECV might also be ascribed to other factors, such as advanced glycation end-product deposition or neovascularization in the myocardial interstitium [38]. These processes may also not cause myocardial strain abnormalities before interstitial fibrosis occurs. Unfortunately, we could not perform histological biopsy analyses to validate the myocardial interstitial components. Moreover, similar to the present study, Vasanji et al. [13] demonstrated the same T1 mapping and myocardial strain findings in T1DM patients. These authors indicated that an increased LV twist function was associated with an elevated myocardial ECV. Therefore, myocardial extracellular matrix expansion may contribute to an enhanced LV twist function, which compensates for the systolic function before the decrease in the LV systolic strain. However, defining the relationships among T1 mapping indicative of interstitial expansion, myocardial systolic strain, and twist function requires more investigation in the future.

In our study, the existence of a significant correlation between the HbA1c level and native T1 or ECV suggests that myocardial interstitial expansion might be ascribed to poor glycemic control. An in vivo animal study demonstrated that hyperglycemia directly led to cardiomyocyte apoptosis by activating reactive oxygen species (ROS) production [43]. ROS generation can induce the formation of advanced glycation end products, which play an important role in collagen accumulation and cardiomyocyte death and ultimately lead to myocardial fibrosis [44]. Furthermore, a greater myocardial ECV was correlated with a greater HbA1c level in obese diabetic adolescents, which was consistent with our results [16]. Two separate studies have reported a negative association between HbA1c and myocardial systolic strain or diastolic function in patients with T2DM [17, 45]. These findings indicate that chronic hyperglycemia has an adverse effect on myocardial interstitial matrix expansion and early heart function abnormalities. Moreover, our findings demonstrated that ACEI treatment was significantly related to a lower myocardial ECV. This result suggests that ACEI treatment appears to exert a protective or reversible effect on myocardial interstitial fibrosis, which is also in agreement with previous studies indicating that ECV and systolic function are ameliorated in diabetic patients by ACEI treatment [17, 37]. Similarly, a decrease in the myocardial collagen volume fraction in response to ACEI treatment has been detected by endomyocardial biopsy in hypertensive patients [46, 47]. Nonetheless, whether ACEI can prevent, delay, or reverse myocardial fibrosis requires further study to determine whether there is a correlation among the duration of ACEI treatment, the order of ACEI treatment and DM diagnosis, and ECV values. T1 mapping to evaluate the effect of medication on the myocardial ECV requires further longitudinal research. Additionally, our findings indicated that an increased myocardial ECV was associated with a lower HCT value in diabetic patients. This result is reasonable because the HCT value is part of the ECV calculation: ECV = (1-hematocrit) × Δ R1_myocardium_/Δ R1_blood_, where R1 = 1/T1 [48]. However, to our knowledge, there are no studies demonstrating that the myocardial interstitial matrix expansion is associated with the HCT value. Whether the myocardial interstitial matrix expansion is associated with lower HCT requires further study.

The present study has several limitations. First, our sample size was small. Second, although previous histological studies demonstrated that the myocardial ECV exhibited significant correlation with the myocardial collagen volume fraction [34], confirming that the increased myocardial ECV in our study was caused by interstitial fibrosis through an endomyocardial biopsy was impossible. Third, our enrolled patients with well-controlled hypertension and controls without hypertension might have led to a bias. Although our subjects included some diabetic patients complicated with hypertension that affected myocardial structure and function, we excluded poorly controlled hypertension according to the above exclusion criteria. Additionally, a difference in blood pressure was not observed between the diabetic patients and healthy controls and a multivariable analysis was performed to exclude this confounding factor. Fourth, we did not use computed tomography angiography or coronary angiography to eliminate volunteers with coronary artery disease (CAD). However, electrocardiography, MRI cine and delayed gadolinium enhancement imaging were performed to exclude overt CAD. Furthermore, an invasive examination was inapplicable for asymptomatic individuals. Fifth, the fasting plasma glucose and hemoglobin A1C levels of the controls were not measured at the time of CMR. However, we obtained a carefully detailed medical history for the controls and checked their medical examination reports within a year of enrollment to ensure that our controls met the inclusion criteria. Sixth, different acquisition protocols are available for T1 mapping, including the modified Look-Locker inversion recovery, saturation recovery single shot acquisition (SASHA), and shortened MOLLI sequence protocols. The MOLLI sequence was selected due to its excellent precision and good reproducibility, as reported in the literature [49].

## Conclusions

In conclusion, our study demonstrates that an increased native myocardial T1 value and ECV can be detected before myocardial systolic strain abnormalities have developed in patients with T2DM. The HbA1c level is a determinant of increased native T1 and ECV indicative of myocardial interstitial expansion, and the amelioration of myocardial interstitial matrix expansion might be associated with ACEI treatment. The increased myocardial ECV was associated with a lower HCT value in diabetic patients. In future research, the effect of the degree of glycemic control and medication on myocardial interstitial expansion warrants a detailed longitudinal, large-scale study. Myocardial T1 mapping might have the potential to quantify the severity of early diabetic myocardial abnormalities and monitor the progress of medication treatment.

## Abbreviations

CMR: cardiovascular magnetic resonance
ECV: extracellular volume fraction
STE: speckle tracking echocardiography
T2DM: type 2 diabetes mellitus
LGE: late gadolinium enhancement
HCT: hematocrit
HbA1c: hemoglobin A1c
BUN: blood urea nitrogen
HDL-C: high-density lipoprotein cholesterol
LDL-C: low-density lipoprotein cholesterol
b-SSFP: balanced steady-state free procession
MOLLI: modified Look-Locker inversion recovery
SASHA: saturation recovery single shot acquisition
PSIR: phase sensitive inversion recovery
LVEDV: left ventricular end-diastolic volume
LVESV: left ventricular end-systolic volume
LVSV: left ventricular stroke volume
LVEF: left ventricular ejection fraction
LVM: left ventricular mass
LAV: left atrial volume
GRS: global radial strain
GCS: global circumferential strain
GLS: global longitudinal strain
ACEI: angiotensin-converting enzyme inhibitor
ICC: intra-class correlation coefficient
ROS: reactive oxygen species
CAD: coronary artery disease
